# Osteogenic Potential of Magnesium Oxide Nanoparticles in Bone Regeneration: A Systematic Review

**DOI:** 10.7759/cureus.55502

**Published:** 2024-03-04

**Authors:** Sankari Malaiappan, Johnisha Harris

**Affiliations:** 1 Department of Periodontics, Saveetha Dental College and Hospitals, Saveetha Institute of Medical and Technical Sciences, Saveetha University, Chennai, IND

**Keywords:** periodontal bone tissue regeneration, bone regeneration, barrier membrane, hydrogel, membrane, scaffold, magnesium oxide nanoparticles, intrabony defects

## Abstract

Guided bone regeneration (GBR) plays a crucial role in the augmentation of alveolar bone, especially in cases of dental implants. The main principle behind using membranes in guided tissue regeneration (GTR) is to prevent epithelial downgrowth as well as connective tissue on the root surface. However, the membranes lack some major properties, such as osteogenic and antimicrobial properties. Magnesium (Mg) is one of the biodegradable materials that is gaining interest because of its favourable mechanical properties and biocompatibility. It also possesses pro-osteogenic properties and significant inhibition of biofilm formation and maturation. These features have attracted increasing interest in using magnesium oxide nanoparticles in GBR membrane applications. This systematic review assesses the osteogenic potential of magnesium oxide nanoparticles in periodontal bone regeneration. The literature search used PubMed, PubMed Central, Medline, and Cochrane databases to examine systematic reviews published till March 2023. Seven articles were included based on the selection criteria. We included all in vitro and in vivo clinical studies based on the osteogenic potential of magnesium oxide nanoparticles in periodontal bone regeneration. The seven studies provided evidence that magnesium oxide nanoparticles, when incorporated in any substrate, showed higher osteogenic potential in terms of higher alkaline phosphatase levels, bone volume fraction, and bone mineral density. The optimum concentration of magnesium oxide can be an ideal additive to various substrates to promote bone regeneration. Because most of the studies were conducted on calvarial defects, further studies should focus only on bone regeneration related to periodontal regeneration.

## Introduction and background

Periodontitis is a chronic inflammatory disease that is mainly characterized by the destruction of the periodontal tissues and the underlying bone. The destruction of the alveolar bone is irreversible and eventually leads to tooth loss [1]. Patients with periodontitis usually report the chief complaints of bleeding gums, tooth mobility, tooth migration, etc. The reason behind tooth mobility is the loss of periodontal tissue and bone, which is only evident radiographically [2]. This process is primarily the result of the activation of osteoclastogenesis. In an attempt to halt the progression of the disease, periodontal therapy is suggested. Periodontal therapy results in repair by the formation of collagenous scar tissue with the migration of the junctional epithelium apically between the connective tissue and the radical surface [3]. This process fails to restore the form and function of lost structures. Hence, the current advancements aim primarily to achieve regeneration in the treatment of intrabony defects, which are described according to the relation of crestal bone to the base of the defect. They are also known as vertical defects, which are further classified as one-walled, two-walled, or three-walled defects [4]. These defects, especially three-walled defects, can be treated with bone grafts and membranes to achieve regeneration. Guided bone regeneration is a regenerative procedure that uses guided bone regeneration membranes (GBRMs), which may or may not require bone substitutes, depending on the morphology of the osseous defect [5]. These membranes prevent the infiltration of fibrous connective tissue and promote bone healing and regeneration [6]. GBRMs may be of two types, resorbable and nonresorbable. The latter has the advantages of strong physical properties and efficiency as a barrier, but the major disadvantage is the second surgery needed to remove the membrane. To avoid this second surgery, absorbable membranes were developed. These membranes can be natural or synthetic polymers. Although conventional collagen or cellulose membranes prevent the downgrowth of the epithelium, connective tissue, and fibroblasts, they still lack osteogenic and antimicrobial properties [7].

To overcome this lack of osteogenic properties, various inorganic substances have been studied to incorporate into these membranes. Among the inorganic compounds, magnesium oxide nanoparticles have gained much attention in the field of tissue engineering [8], primarily due to their ability to accelerate osteogenic differentiation of osteoblasts and antibacterial activity. Magnesium (Mg) ions also act as coenzymes in various metabolic pathways of the body. Magnesium alloys also produce hydrogen gas as an end product, which accelerates the accumulation of magnesium ions in body fluids, thereby inhibiting osteoblast activity. Thus, the concentration of magnesium used also significantly affects differences in tissue engineering. Magnesium oxide nanoparticles possess biodegradability, minimal toxicity, pro-osteogenic activity, and antimicrobial activity, making them an ideal component in guided tissue regeneration membranes [9]. Magnesium has been shown to be degradable in aqueous solutions and biocompatible in natural doses. However, necessary steps must be taken to prevent rapid degradation and enhance its biocompatibility [10]. We conducted this systematic review to evaluate the osteogenic potential of magnesium oxide nanoparticles in bone tissue regeneration.

## Review

Aim

The aim of this systematic review is to evaluate the osteogenic potential of magnesium oxide (MgO) nanoparticles in bone regeneration in the treatment of intrabony defects.

Materials and methods

Protocol

**Figure 1:**
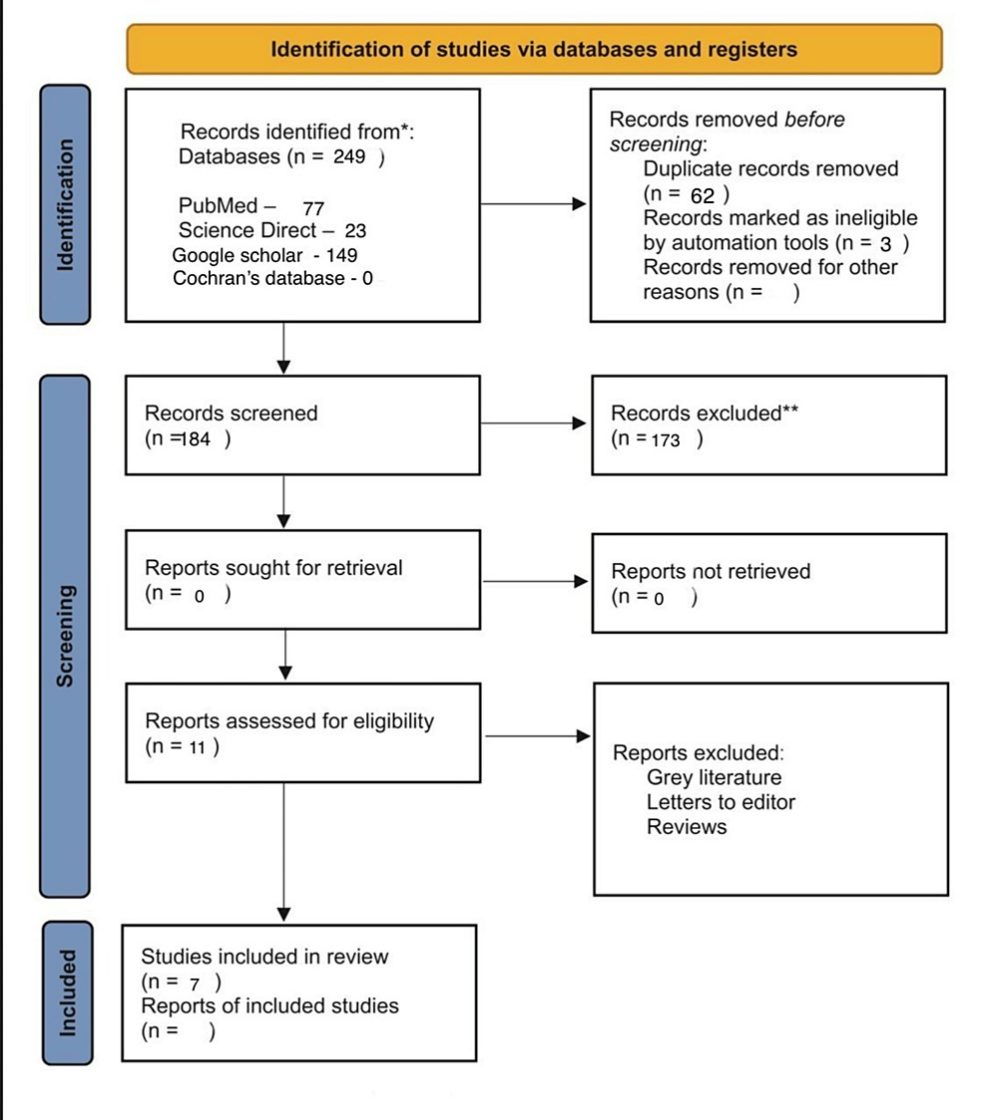
PRISMA chart PRISMA: Preferred Reporting Items for Systematic Reviews and Meta-Analyses.

We conducted this systematic review according to the Preferred Reporting Items for Systematic Reviews and Meta-Analyses (PRISMA) guidelines (Figure 1). This study was registered in the International Prospective Register of Systematic Reviews (PROSPERO; registration number: CRD42023450619).

Focus Question

Do magnesium oxide nanoparticles have osteogenic potential to promote bone regeneration?

PICO Analysis

The PICO (population, intervention, comparison, and outcome) analysis used was as follows: population - intrabony defects; intervention - magnesium oxide nanoparticles-based scaffold or membrane in the treatment of intrabony defects; comparison - membranes or scaffolds without magnesium oxide nanoparticles; outcome - bone regeneration.

Search Strategy

We conducted a literature search in PubMed, PubMed Central, Medline, and the Cochrane Database of Systematic Reviews to obtain studies published till March 2023. We selected articles based on the selection criteria.

MeSH Terms

(((Magnesium oxide [Text Word]) AND (nanoparticles [Text Word])) AND (bone regeneration [Text Word])) OR (Bony regeneration [Text Word]) "magnesium oxide nanoparticles" OR "magnesium oxide nanoparticles MgO NPs") AND ("hydrogel" OR "scaffold" OR "barrier membrane" OR "bone cement") AND ("bone regeneration" OR "bone tissue regeneration").

Risk of Bias Assessment

Risk of bias assessment was done using the QUIN (Quality Assessment Tool for In Vitro Studies) tool for in vitro studies and the SYRCLE (Systematic Review Centre for Laboratory Animal Experimentation) tool for animal studies, as mentioned in Tables 1, 2. Two authors evaluated all the required parameters for risk of bias assessment. The assessment was done based on the following parameters: random sequencing, implementing single operator protocol, control group, blinding the reviewer, sample standardization, evaluation of failures, and using materials in accordance with the instructions of the manufacturer. After examination, based on the number of “YES” responses, the risk of bias was classified.

**Table 1:** Risk of bias assessment using the SYRCLE tool SYRCLE: Systematic Review Centre for Laboratory Animal Experimentation.

S. No.	Signalling question	Chen et al. (2021) [11]	Nan et al. (2022) [12]	Xing et al. (2020) [13]	Yuan et al. (2019) [14]	Peng et al. (2021) [15]	
1	Was the allocation sequence adequately generated and applied?	No	Unclear	Unclear	Unclear	Unclear	Unclear
2	Were the groups similar in baseline or were they adjusted for confounders in the analysis?	Yes	Yes	Yes	Yes	Yes	Low bias
3	Was the allocation adequately concealed?	Unclear	Unclear	Unclear	Unclear	Unclear	Unclear bias
4	Were the animals randomly housed during the experiment?	Unclear	Unclear	Unclear	Unclear	Unclear	Unclear bias
5	Were the caregivers and or investigators blinded from knowledge of which intervention each animal received during the experiment?	No	No	No	No	No	Low bias
6	Were the animals selected at random for outcome assessment?	Yes	Yes	Yes	Yes	Yes	Low bias
7	Was the outcome assessor blinded?	Unclear	Unclear	Unclear	Unclear	Unclear	Unclear bias
8	Were incomplete outcome data adequately addressed?	Yes	Yes	Yes	Yes	Yes	Low bias
9	Are reports of the study free of selective outcome reporting?	Yes	Yes	Yes	Yes	Yes	Low bias
10	Was the study apparently free of other problems that could result in a high risk of bias?	Yes	Yes	Yes	Yes	Yes	Low bias
	Risk of bias	Medium bias	Medium bias	Medium bias	Medium bias	Medium bias	

**Table 2:** Risk of assessment using the QUIN tool QUIN: Quality Assessment Tool for In Vitro Studies.

S. No.	Criteria	Shen et al. (2019) [16]	Wang et al. (2021) [17]
1	Clearly stated aims/objectives	2	1
2	Detailed explanation of sample size calculation	1	0
3	Detailed explanation of the sampling technique	0	0
4	Details of the comparison group	2	1
5	Detailed explanation of the methodology	2	2
6	Operator details	0	0
7	Randomization	0	0
8	Method of measurement of outcome	2	2
9	Outcome accessor details	2	0
10	Blinding	0	0
11	Statistical analysis	2	2
12	Presentation of results	2	2
	Total score	15	10
	Kappa value	0.62	0.42
	Risk of bias	Medium risk	High risk

Data Extraction

Data from the included studies were collected independently with the aid of two reviewers using a customized data extraction and were entered into an electronic spreadsheet, including the name of the authors, year of publication, total number of participants, and outcome measures (Table 3).

**Table 3:** Characteristics of included studies HA: hydroxyapatite; MgO: magnesium oxide; HA/MgO-H): hydroxyapatite/magnesium oxide-nanocrystal hybrid hydrogel; Cys-H: PGA-Cys hydrogel; PGA-Cys: cysteine-modified gamma-polyglutamic acid; BMD: bone mineral density; CON: control; TaNPs: tantalum nanoparticles; PCL: polycaprolactone; Ta: tantalum; BMSCs: bone marrow mesenchymal stem cells; SF/PCL: silk fibroin/polycaprolactone; BMP2: bone morphogenetic protein 2; OCN: osteocalcin; ALP: alkaline phosphatase; RUNX2: runt-related transcription factor 2; PLGA: poly(lactide-co-glycolide); MgCO3: magnesium carbonate; PMg-III: biodegradable microsphere; BV: bone volume; TV: total volume; Mg2+: magnesium ions; EdU: 5-ethynyl-2′-deoxyuridine; RT-qPCR: real-time quantitative polymerase chain reaction; hPDLSCs: human periodontal ligament stem cells; ANOVA: analysis of variance; GTR: guided tissue regeneration; PCL-PEG-PCL: polycaprolactone-co-poly(ethylene glycol)-co-polycaprolactone; T-MgO: silane-coated MgO nanoparticles; C/M-T1: newly prepared nanocomposite; C/M-T2: nanocomposite with excessive magnesium ionic microenvironment; ECM: extracellular matrix; ICP-AES: inductively coupled plasma atomic emission spectroscopy; PLA: poly-l-lactic acid; PEG: polyethylene glycol; ARS: Alizarin red staining; SD: Sprague-Dawley.

S. No.	Author	Study design	Aim	Material used	Methodology	Outcomes	Conclusion
1	Chen et al. (2021) [11]	In vitro & in vivo	To evaluate the bone repair ability of HA(MgO-H) scaffold in diabetic rats	HA(MgO-H)	n = 30 diabetic rats anaesthetised with 10% chloral hydrate. A defect of 3 mm diameter & depth was created using a drill in the femur bone. Five groups (6 rats for each group): (1) control group; (2) Cys-H group; (3) HA-H group; (4) MgO-H group; (5) HA/MgO-H group	BMD value in the HA/MgO-H group was significantly higher than that in the CON and Cys-H group (p < 0.01).	HA/MgO nanocrystal hybrid hydrogel is a promising strategy to rapidly achieve bone repair for people with type 1 diabetes
2	Nan et al. (2022) [12]	In vitro & In vivo	To check for the osteogenic and angiogenic ability of tantalum nanoparticles (TaNPs) and nanoscale magnesium oxide (MgO) infused nanofiber periosteum replacement based on poly-ε-caprolactone (PCL)	PCL/Ta with MgO	n = 12 (3 rats per group). Four groups: (1) no treatment; (2) pure PCL membrane; (3) PCL/Ta without MgO; (4) PCL/Ta with MgO. A defect 4 mm in diameter was then made by trephine on either side of the coronal suture. Follow-up after 4 weeks	Bone volume fraction pure PCL membranes - 14.36 ± 0.75%. PCL/Ta group - 29.07 ± 1.80%. PCL/Ta/MgO - 41.76 ± 6.40%	The presence of TaNPs and the sustained release of Mg2+ had an inductive effect on the osteogenic differentiation of BMSCs and an angiogenic effect
3	Xing et al. (2020) [13]	In vitro & in vivo	The release kinetics of Mg2+ and the effects of magnesium on scaffold morphology, and cellular behaviour were studied	Membrane made of magnesium oxide (MgO) nanoparticles into silk fibroin & polycaprolactone (SF/PCL)-blend scaffolds	n = 24. Three groups (8 rats each): (1) control; (2) SF/PCL group; (3) SF/PCL/MgO group. Full-thickness calvarial defects with diameters of 5 mm were created symmetrically on both sides of the middle ridge by using a trephine	BMP-2, OCN, ALP, and RUNX-2 expression were evaluated. SF/PCL/MgO - on day 7, BMP2 - 6.4-fold increased expression; OCN - 4.4-fold; ALP - 3.1-fold; RUNX2 - 4.5-fold higher expression	The Mg2+- containing SF/PCL scaffolds promoted adherence and bone regeneration. Magnesium-containing SF/PCL composite membrane can be used as a potential candidate for bone regeneration therapy
4	Yuan et al. (2019) [14]	In vitro & animal study	To study the release behaviours of Mg2+ from the microspheres, which were correlated with their abilities to induce mineral depositions and regulate cell activities such as cell migration, proliferation, and osteogenic differentiation of seeded BMSCs	PLGA/MgO/MgCO3 (PMg) microspheres	n = 30 female SD rats (5-6 weeks old), 8 mm calvarial defect model. Three groups (10 rats for each group): (1) control group; (2) PLGA group; (3) PMg-III group. Briefly, the rats were shaved and anaesthetized with chloral hydrate (0.5 mL/250 g). The 8 mm defect was created at the central region of the calvarium with a trephine bur. PLGA or PMg-III microspheres were filled into the defects, the overlying tissue was closed with surgical staples	Follow-up: 16 weeks postoperatively. Unfilled - BV/TV (4.6 ± 0.7 %), BMD (45.7 ± 23.2 mg/cm3). PLGA - BV/TV (8.1 ± 2.5%), BMD (124 ± 35.8 mg/cm3). PMg-III - BV/TV (32.9 ± 5.6%) and BMD (325.7 ± 20.2 mg/cm3)	These PMg microspheres provided feasibility to regulate biomineralization, cell migration, and osteogenic differentiation, as well as antibacterial activity in a wide range of Mg2+ ions
5	Peng et al. (2021) [15]	In vitro study	To check the osteogenic & antimicrobial properties of cellulose nanofiber incorporated with MgO	Coaxial-MgO	Polycaprolactone/gelatin nanocellulose membranes were prepared by the electrospinning method. Cell experiment, cell proliferation assay, EdU staining assay, cell attachment assay, ALP activity and staining, Alizarin red staining, RT-qPCR antibacterial activity	Coaxial-MgO exhibited optimal adhesion and proliferation of hPDLSCs. Expression of ALP activity and formation of mineralized nodules of hPDLSCs treated with coaxial-MgO were higher. Improved the expression of osteogenic-related genes, such as ALP and Runx2. Decrease of colonies in the Coaxial-MgO group. Statistical analysis: one-way ANOVA followed by Tukey’s post hoc analysis	MgO nanoparticles-incorporated PCL/gelatin nanocellulose membranes fabricated by coaxial electrospinning with excellent osteogenic and antibacterial properties may be used as GTR membranes
6	Shen et al. (2019) [16]	In vitro & animal study	To demonstrate a tunable magnesium ionic (Mg2+) microenvironment in bony tissue that can significantly induce bone defect repair	PCL-PEG-PCL/T-MgO 10 g of copolymer + MgO in 4 different concentrations	Cell proliferation osteogenic differentiation, histological analysis, implantation within the lateral epicondyle defect, and intraperitoneal injections of calcein green and xylenol orange were performed at 4 and 6 weeks post-surgery	BMDs control group = 0.65512 ± 0.0183; PCL group = 0.70271 ± 0.0130; copolymer group = 0.74598 ± 0.0373; C/M-T2 group = 0.71207 ± 0.0074; ALP assay = day 7; PCL = 568.1 ± 36.9 U/mg; copolymer = 893.1 ± 25.8 U/mg; C/M-T1 groups = 1024.8 ± 199.3 U/mg; C/M-T2 group = 162.2 ± 17.7 U/mg	The concentration of magnesium ions in the local tissue microenvironment has to be carefully designed, to print 3D printed PCL-PEG-PCL/T-MgO scaffold for bone tissue repair
7	Wang et al. (2021) [17]	In vitro study	To fabricate porous nanofibrous scaffolds structurally similar to native extracellular matrix incorporated with MgO nanoparticles	PLA/gelatin/MgO	Atomic emission spectroscopy to measure the Magnesium ion release	Magnesium release mainly occurred within the first two days as quantified by ICP-AES. This indicates that the hydrolysis of MgO nanoparticles partially neutralizes the acidic degradation products of PLA	MgO nanoparticle-incorporated scaffold exhibits nanofibrous ultrastructure with open pores, similar to native ECM. Good cytocompatibility and elevates osteogenic differentiation of pre-osteoblasts

Results

The initial search yielded 249 entries in PubMed, Google Scholar, Cochrane, and manual search. After the exclusion of all the duplicate articles, a total of 11 articles were selected. Exclusion was done based on articles/full-text screen and inclusion and exclusion criteria. After data extraction, a total of seven articles were selected for this review. From these articles, data extraction was done, and study characteristics were tabulated (Figure 1).

The articles were assessed and segregated. The data were collected from the included studies with the help of different aspects of the following structured question: “Do magnesium oxide nanoparticles have osteogenic potential in bone regeneration?"

According to the above articles, magnesium oxide nanoparticles have osteogenic potential to aid in bone regeneration. The study by Chen et al. was similar to previous studies with four times more alkaline phosphatase (ALP) activity and calcium deposition in the hydroxyapatite/magnesium oxide-nanocrystal hybrid hydrogel (HA/MgO-H) group than in the control group. The test group also showed 1.2 times higher bone volume/total volume (BV/TV) in the HA/MgO-H group than in the control group [11]. The study by Nan et al. reported maximum Ca nodule formation in polycaprolactone (PCL)/tantalum (Ta)/MgO membranes, which is consistent with the previous results. It validated the ability of Ta to induce osteogenesis and MgO to enhance osteogenic differentiation. The authors performed an ALP assay because it is a reliable marker to prove early osteogenic activity. PCL/Ta/MgO group showed the highest ALP activity compared to both the other groups on day seven [12]. Xing et al. studied the characteristics of magnesium oxide nanoparticles that were embedded in the polycaprolactone scaffolds fabricated through electro-spinning and placed in calvarial defects. During histological analysis, it was found that more mature collagen was found in silk fibroin (SF)/PCL/MgO groups than in the control group. SF/PCL/MgO group also showed the highest ALP activity, osteogenic activity, and mineralized modules [13]. Yuan et al. compared the bone volume fraction and bone mass density among the control group, which is unfilled with MgO-containing microspheres filled defects at the end of 12 weeks [14]. They showed a mean difference of 28.3 ± 4.9% bone volume fraction and 280 ± 3.2 mg/cm3 bone mass density [14]. Another study by Peng et al. evaluated the osteogenic potential of magnesium-incorporated composite scaffold in terms of ALP levels and found that staining intensity was higher in the MgO group with MC3T3-E1 cells than those without MgO on the 14th day. On the 21st day, more calcium nodules were formed in the MgO group than in the control group [15]. Shen et al. studied the osteogenic potential among three groups, out of which groups 2 & 3 contained magnesium whereas group 1 lacked magnesium. The results of the ALP assay showed that the osteogenic differentiation and bone regeneration were at the peak in group 2 compared to group 3 with excessive magnesium [16]. The study by Wang et al. revealed a morphological change in the fibroblasts from a round shape on day four to a spindle shape on day seven in poly-l-lactic acid (PLA)/gelatin/MgO scaffold, whereas, in PLA/gelatin scaffold, the cells were still in a round shape between days four and seven. Similarly, ME3T3-E1 cells exhibited higher ALP activity in the PLA/gelatin/MgO scaffold than in the PLA/gelatin scaffold at the end of the 14th day [17].

Summary Synthesis

Consequently, we provided a narrative synthesis of the findings, summarizing the key results of each study and identifying any patterns or trends that emerged. Although we were not able to perform a meta-analysis, our narrative synthesis provided a qualitative summary of the available evidence, allowing us to draw meaningful conclusions to implement in clinical practice.

Discussion

Periodontitis, which is mainly characterized by the inflammation and destruction of supporting periodontal tissues, when left untreated, leads to severe bone loss, causing osseous defects, eventually leading to tooth loss. These osseous defects can be treated in two ways, either resective osseous surgery or regenerative periodontal therapy, depending on the pattern of bone loss. Regenerative periodontal therapy requires not only bone grafts but also membranes to prevent epithelial downgrowth [18]. Such membranes should possess certain properties like osteogenic or osteoinductive potential, which aids in bone regeneration. Since periodontitis is a polymicrobial disease, the membrane used to treat periodontal osseous defects is also expected to possess antimicrobial properties to fight against periodontal pathogens [19].

This systematic review mainly focuses on assessing the existing scientific evidence on the osteogenic potential of magnesium oxide nanoparticles to aid bone regeneration. Unfortunately, statistical analysis was not possible due to the heterogeneity of the included studies. However, a rise of publication bias might be possible since the search was restricted to only the English language. Unpublished data and commercial interests were not explored. An attempt was made to pool the data; however, it could not be done because of the variable outcome measures and follow-up periods. Differences in the ALP levels, bone volume fraction, and bone mass density were significant. A mean difference of 11.69 ± 4.6% was found between membranes without nanoparticles and MgO-loaded nanoparticles during the follow-up after four weeks. ALP levels were 3.1-fold higher in MgO-containing scaffold at the end of day seven, thereby indicating that it promoted cell adherence and bone regeneration. Yuan et al. compared the bone volume fraction and bone mass density between the control group, which is unfilled with MgO-containing microspheres filled defects at the end of 12 weeks. They showed a mean difference of 28.3 ± 4.9% bone volume fraction and 280 ± 3.2 mg/cm3 bone mass density [14].

Shen et al., in their study on the addition of magnesium oxide in four different concentrations, concluded that only optimum concentrations of MgO favour osteogenic differentiation. This was done after four and six weeks post-surgery. They showed a mean difference of 456 ± 3.6 U/mg [16]. Though magnesium oxide showed better results than the control group, the addition of excess magnesium oxide nanoparticles led to decreased ALP levels. The study by Wang et al. tested the effects of the magnesium oxide microenvironment on the surrounding tissues indicating the highest level of osteogenic differentiation of pre-osteoblasts [17]. Even though the overall results show that magnesium oxide nanoparticles influence increased bone regeneration, compared to the control group, the inconsistent follow-up period and outcome variables still question the reliability of the same under clinical use in humans [18]. All in vivo studies have been carried out in calvarial defects and hence other clinical parameters like probing pocket depth, clinical attachment loss, and bleeding index could not be measured like periodontal intrabony defects [20]. Based on the results of the included studies, most studies revealed that magnesium oxide has a positive influence on bone regeneration. However, the selected studies do not completely satisfy the requirements of study quality. Study designs, methods of randomization, allocations concealment, and examiner blinding could not be assessed sufficiently. In some studies, the validity of outcomes and estimation was considered to be unclear or even inconclusive [10]. Failure to implement these methods would lead to the risk of bias in trial outcomes. Based on the details, data obtained from low-quality studies cannot be used as evidence.

To enhance evidence, well-documented clinical trials on magnesium oxide nanoparticle-based membranes are required. Heterogeneity was observed when the selected studies were reviewed. No standardized protocol was followed by all the studies to prevent bias. Information regarding the fabrication of the membrane and the steps in the fabrication were not mentioned in all the studies. Only a detailed description of the results was provided. In the current systematic review, the inclusion of certain parameters was recommended to meet the inclusion criteria. In summary, the results provide evidence for the use of magnesium oxide nanoparticles in the treatment of periodontal intrabony defects and their role in achieving bone regeneration. The dental literature provides sufficient strong evidence that magnesium oxide is beneficial for bone regeneration. Further clinical trials should be carried out to find its efficiency in bone regeneration in humans under a clear standardized protocol.

Limitations

Though magnesium-based biomaterials have proven to be efficient in anti-inflammatory properties, enhanced mineralization rate, and improved repair of bone tissues, high concentrations of Mg2+ have detrimental effects on human osteoblast differentiation, osseous metabolism, and homeostasis, which may lead to bone mineralization defects and even contribute to bone disease [21].

Clinical significance

Based on the results of the systematic review, it is evident that magnesium oxide nanoparticles possess osteogenic potential to enhance bone regeneration. Hence, this property can be taken into consideration in the development of membranes, which can further be used in clinical trials, especially in cases of ridge augmentations where bone regeneration is most essential.

## Conclusions

This systematic review led us to conclude that magnesium oxide nanoparticles, when incorporated in a substrate, showed higher osteogenic potential in terms of higher alkaline phosphatase levels, bone volume fraction, and bone mineral density. The optimum concentration of magnesium oxide can prove to be an ideal additive to various substrates to promote bone regeneration. Because most of the studies were conducted in calvarial defects, further studies should focus only on the aspect of bone regeneration in terms of periodontal regeneration. Further studies are needed to assess the efficiency of magnesium oxide nanoparticles in bone regeneration using clinical trials.
